# Evaluating the Bidirectional Causal Effects of Alzheimer’s Disease Across Multiple Conditions: A Systematic Review and Meta-Analysis of Mendelian Randomization Studies

**DOI:** 10.3390/ijms26083589

**Published:** 2025-04-10

**Authors:** Haoning Zhu, Huitong Ni, Qiuling Yang, Jiaqi Ni, Jianguang Ji, Shu Yang, Fu Peng

**Affiliations:** 1 Key Laboratory of Drug-Targeting and Drug Delivery System of the Education Ministry and Sichuan Province, Sichuan Engineering Laboratory for Plant-Sourced Drug, Sichuan Research Center for Drug Precision Industrial Technology, West China School of Pharmacy, Sichuan University, Chengdu 610041, China; zhuhn_2019@163.com (H.Z.); nihuitong520@163.com (H.N.); jiaqini@scu.edu.cn (J.N.); fujing126@yeah.net (F.P.); 2 Institute of Materia Medica, Chinese Academy of Medical Sciences and Peking Union Medical College, Beijing 100050, China; qlyang@imm.ac.cn; 3 Faculty of Health Sciences, University of Macau, Macau SAR, China

**Keywords:** Mendelian randomization, Alzheimer’s disease, systemic disease phenotype, causal relationship, system evaluation

## Abstract

This study systematically evaluates and meta-analyzes Mendelian randomization studies on the bidirectional causal relationship between Alzheimer’s disease (AD) and systemic diseases. We searched five databases, assessed study quality, and extracted data. Diseases were classified using ICD-11, and the meta-analysis was performed with RevMan 5.4. A total of 56 studies identified genetic links between AD susceptibility and systemic diseases. Notably, genetic proxies for hip osteoarthritis (OR = 0.80; *p* = 0.007) and rheumatoid arthritis (OR = 0.97; *p* = 0.004) were inversely associated with AD risk, while gout (OR = 1.02; *p* = 0.049) showed a positive association. Genetic liability to depression (OR = 1.03; *p* = 0.001) elevated AD risk, and AD genetic risk increased susceptibility to delirium (OR = 1.32; *p* = 0.0005). Cardiovascular traits, including coronary artery disease (OR = 1.07; *p* = 0.021) and hypertension (OR = 4.30; *p* = 0.044), were causally linked to a higher AD risk. Other conditions, such as insomnia, chronic periodontitis, migraine, and certain cancers, exhibited significant genetic correlations. Intriguingly, herpes zoster (OR = 0.87; *p* = 0.005) and cataracts (OR = 0.96; *p* = 0.012) demonstrated inverse genetic associations with AD. These findings suggest potential therapeutic targets and preventive strategies, emphasizing the need to address comorbid systemic diseases to reduce AD risk and progression.

## 1. Introduction

As the population ages, the prevalence of dementia is expected to increase dramatically in the coming decades. It is projected that, by 2050, over 115 million people worldwide will have dementia, with Alzheimer’s disease (AD) being the primary cause. AD is a complex progressive neurodegenerative disease that is currently considered the main form of adult dementia. Its clinical manifestations include progressive cognitive decline, memory impairment, emotional disorders, aphasia, language dysfunction, and other symptoms [1,2]. AD dementia may also contribute to or directly cause death [3]. In 2019, AD and other dementias ranked as the seventh leading cause of global death [4]. The economic burden of AD and related dementias is increasing globally, with the global disease burden of AD and related dementias estimated to be USD 2.8 trillion in 2019 based on the Value of Statistical Life [5].

AD is a multifactorial complex disease with a complex pathogenesis. While our understanding of the molecular and biochemical mechanisms underlying AD is expanding, the true causes and pathogenesis remain unknown, complicating the development of innovative therapeutic drugs. With several new small-molecule drugs failing in clinical trials, the rational strategy for developing new AD therapeutic drugs has shifted from a “single-target” approach to a “multi-target” approach, aiming to enhance efficacy while minimizing side effects. Recent studies have demonstrated a causal relationship between multiple diseases and AD [6,7,8]. Therefore, it is important to utilize these findings to develop new drug targets and preventive treatment strategies for AD. Mendelian randomization (MR) is a method that uses genetic variations as instrumental variables to estimate causal relationships between exposure and disease outcomes. Currently, there are a considerable number of studies that have used the MR method to study the relationships between AD and other diseases. However, most of these studies only explore the association of a single disease with AD, lacking systematic analysis and facing issues such as unclear causal relationships, vague quality, and data source overlap, making it difficult to provide a reference for subsequent AD research. Based on the multi-target strategy in current AD drug development, analyzing diseases that may be associated with an increased risk of AD can provide a theoretical basis for drug target selection and guide hypothesis-driven experimental validation, thereby accelerating the drug development process for AD.

To comprehensively evaluate and summarize the causal effects between Alzheimer’s disease (AD) and various other diseases, this article reviews published Mendelian randomization (MR) studies on the relationship between AD and other disease phenotypes. By examining the genetic evidence for causal relationships between AD and multiple other diseases, we aim to explore the mutual mechanistic interactions between AD and these conditions, offering new insights for drug development and preventive strategies for AD.

## 2. Materials and Methods

### 2.1. Literature Retrieval Strategy

The databases included PubMed, EMBASE, CNKI (China National Knowledge Infrastructure), the VIP Chinese Science and Technology Journal Database, and the Wanfang Medical Database. A combination of subject terms and free terms was used to search the literature using the MR method to study the relationship between AD and disease phenotypes. Search terms in Chinese and English included Mendelian randomization, Alzheimer’s disease, and relevant Mesh terms. The search time frame was from database creation to 19 January 2024. There were no restrictions on the document type or language during the search.

### 2.2. Criteria for Inclusion and Exclusion of Literature

#### 2.2.1. Inclusion Criteria

The inclusion criteria were as follows:

(1) Published articles exploring the causal relationship between AD and multiple diseases using MR methods, including unidirectional or bidirectional MR studies;
(2) Studies using instrumental variable methods (IVW) to report the association between AD and multiple diseases in the form of odds ratios (ORs) and 95% confidence intervals (95% CIs).

#### 2.2.2. Exclusion Criteria

The exclusion criteria were as follows:

(1) Articles without sufficient estimated information, those that were irrelevant to the topic, or those on diseases not standardized in ICD-11 were excluded;
(2) Case reports, narrative reviews, letters, opinions, foreign-language translations, reviews, incomplete manuscripts, non-original MR studies, and conference abstracts were excluded;
(3) Articles without extractable indicators were excluded;
(4) Articles with disputed results were excluded;
(5) When there were multiple publications based on the same GWAS (same participants), only publications with the largest sample size or the latest published study (if the sample size was the same) were included, without sample size limitations.

### 2.3. Literature Screening and Data Extraction

Articles retrieved using the search strategy were imported into EndNote X9 for duplicate removal. The initial screening of titles and abstracts was conducted by two independent reviewers, and full texts were reviewed to identify studies meeting the inclusion criteria. Any disagreements were resolved through discussion with a third reviewer. Based on a pre-established data extraction table, original information was extracted from the literature. Data extraction was performed by one reviewer using predefined tables and independently verified by two more reviewers. The extracted data included general information (article title, authors, publication year, abstract), study details (exposure and outcome studied, study population, sample size (number of cases and non-cases), exposure GWAS data source, outcome GWAS data source, main causal effect estimation method), and result data (computed OR values, 95% CI, *p*-values). The extracted result data are presented in Table A2.

### 2.4. Literature Quality Evaluation

The quality of MR studies included in this systematic evaluation was assessed using a modified version of the STROBE-MR checklist, which was modified and applied in previously published articles on Mendelian randomization reporting quality approaches [9,10]. This modified tool was used to evaluate reporting quality by converting the scores to percentages, with scores below 80%, between 80% and 90%, and above 90% representing a high, medium, and low risk of bias, respectively. The assessment was conducted by two reviewers, with a third reviewer being consulted to resolve any disagreements or uncertainties.

### 2.5. Statistical Analysis

When multiple MR estimates were available for the same outcome based on non-overlapping samples, meta-analysis was conducted using Review Manager 5.4 software to obtain combined estimates, with the choice of model determined by the I2 value. Heterogeneity among studies was assessed using I2, with values between 25% and 50% considered as mild heterogeneity, values between 50% and 75% as moderate heterogeneity, and values above 75% as severe heterogeneity. A random-effects model was chosen when I2 was greater than 50%, and a fixed-effects model was chosen when I2 was less than or equal to 50%. In the final presentation of results, the forest plot only indicates the Meta combined estimate for that disease.

Sensitivity analyses were not performed because all included studies adhered to uniform methods, including the use of the IVW method for Mendelian randomization, and were based on consistent and reliable data sources, such as large GWAS consortia. Additionally, the MR estimates were derived from non-overlapping datasets, minimizing the risk of bias or inconsistency that would typically warrant sensitivity testing.

### 2.6. Research Plan Description

All MR research data come from large GWAS meta-analyses (consortia), UK biological sample banks, FinnGen studies, the Million Veterans Program, or outcome data from two or more of these sources. All studies have provided results with AD as the exposure or outcome based on the IVW method. All MR studies provided effect estimates with AD as either the exposure or the outcome, calculated using the inverse variance-weighted (IVW) method. The effect measures included odds ratios (ORs) with 95% confidence intervals (CIs), which were used to evaluate the causal relationships between AD and the associated outcomes. In the discussion of the results, the disease was searched in ICD-11, and the largest submenu where the disease is located was selected as the system. For diseases that could not be retrieved from ICD-11, such as metabolic syndrome [11], their MR studies were not included in the evaluation system.

## 3. Results

### 3.1. Literature Search Results

**Figure 1:**
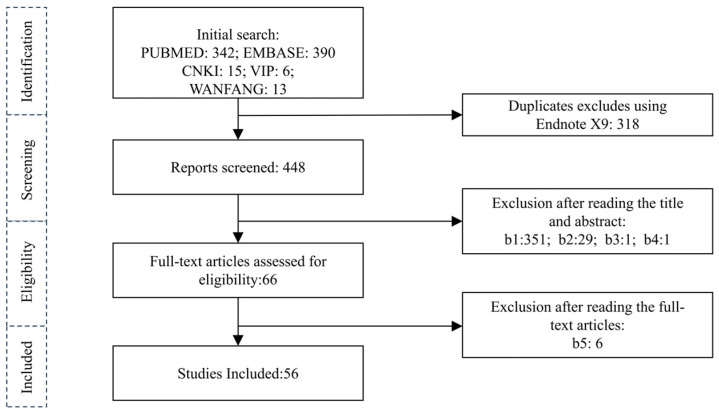
The design overview of this study. b1, Articles without sufficient estimated information, articles unrelated to the topic, and diseases that are not standardized in ICD-11 are excluded; b2, any case reports, narrative comments, letters, opinions, foreign-language translations, reviews, incomplete manuscripts, non-original MR studies, and conference abstracts are excluded; b3, articles without extractable indicators are excluded; b4, articles with disputed results are excluded; b5, when there are multiple publications based on a GWAS (same participants) with the same results, only publications with the largest sample size or the latest published study (if the sample size is the same) are included, without sample size limitations.

Based on the established literature search strategy, the search results are shown in Table A1. A total of 766 articles were retrieved using the MR method to study the causal relationship between AD and various phenotypes. After checking for duplicates using Endnote X9 software, there were 448 remaining articles. After reading the titles and abstracts for initial screening and then reading the entire texts, the articles that could not be obtained or were excluded during screening were eliminated, resulting in a total of 60 articles. The literature screening process is shown in Figure 1.

### 3.2. Basic Characteristics of Included Literature

A total of 60 original studies were included, with a publication period from database creation to 19 January 2024. The basic characteristics of the included literature are shown in Table 1.

### 3.3. Results of Literature Quality Evaluation

A quality evaluation was conducted on the 60 included articles using the modified STROBE-MR checklist [68,69]. A total of 31 articles were assessed as having a low risk of bias, 23 articles were assessed as having a moderate risk of bias, and 6 articles were assessed as having a high risk of bias. The specific evaluation results of all studies are detailed in Table 2.

### 3.4. The Bidirectional Causal Relationship Between AD and Multiple Diseases

**Figure 2:**
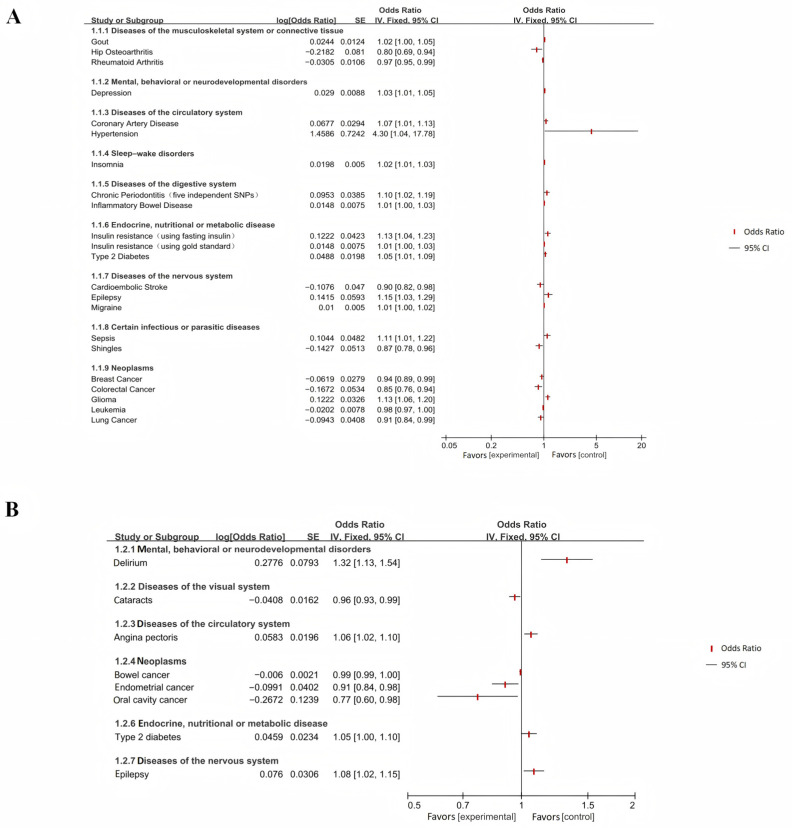
The forest plot of bidirectional causal effects between AD and various systemic diseases (significant results section). (**A**) The results of MR analysis of assessing the effects of various systemic diseases on AD. (**B**) The results of MR analysis of assessing the causal effects of AD on various systemic diseases.

Through a Mendelian randomization study on the bidirectional causal relationship between Alzheimer’s disease (AD) and various systemic disease phenotypes, a systematic evaluation and meta-analysis were conducted. Finally, 60 articles were selected, and the analysis results demonstrated genetically predicted causal links between AD and systemic diseases, with comprehensive data presented in Table 3 and forest plot results illustrated in Figure 2.

**Figure A1:**
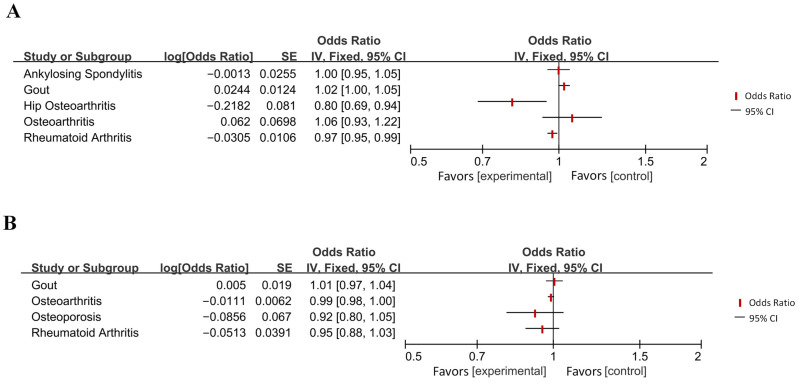
Bidirectional causal effects between AD and diseases of the musculoskeletal system or connective tissue. (**A**) The results of MR analysis of assessing the causal effects of diseases of the musculoskeletal system or connective tissue on AD. (**B**) The results of MR analysis of assessing the causal effects of AD on diseases of the musculoskeletal system or connective tissue.

Among musculoskeletal system or connective tissue diseases, hip osteoarthritis (OR = 0.80; 95% CI: 0.69–0.94; *p* = 0.007) and rheumatoid arthritis (OR = 0.97; 95% CI; 0.95~0.99; *p* = 0.004) presented an inverse association with AD risk, while gout showed a positive association (OR = 1.02; 95% CI: 1.00–1.05; *p* = 0.049) (Figure 2 and Figure A1).

**Figure A2:**
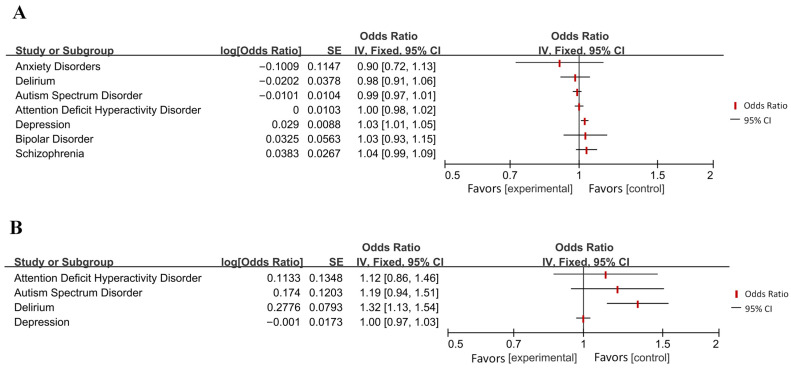
Bidirectional causal association between AD and mental, behavioral, or neurodevelopmental disorders. (**A**) The results of MR analysis of assessing the causal effects of mental, behavioral, or neurodevelopmental disorders on AD. (**B**) The results of MR analysis of assessing the causal effects of AD on mental, behavioral, or neurodevelopmental disorders.

In the category of mental, behavioral, or neurodevelopmental disorders, depression elevated AD risk (OR = 1.03; 95% CI: 1.01–1.05; *p* = 0.001) (Figure 2A and Figure A2). AD increased susceptibility to delirium (OR = 1.32; 95% CI: 1.13–1.54; *p* = 0.0005) (Figure 2B and Figure A2).

**Figure A3:**
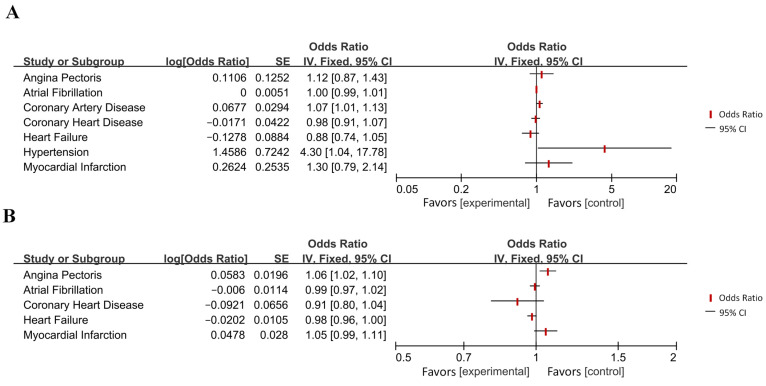
Bidirectional causal association between AD and diseases of the circulatory system. (**A**) The results of MR analysis of assessing the causal effects of diseases of the circulatory system on AD. (**B**) The results of MR analysis of assessing the causal effects of AD on diseases of the circulatory system.

Concerning circulatory system diseases, coronary artery disease (OR = 1.07; 95% CI: 1.01–1.13; *p* = 0.021) and hypertension (OR = 4.30; 95% CI: 1.04–17.78; *p* = 0.044) demonstrated positive causal effects on AD (Figure 2A and Figure A3). AD was associated with a higher risk of angina (OR = 1.06; 95% CI: 1.02–1.10; *p* = 0.003) (Figure 2B and Figure A3).

**Figure A4:**
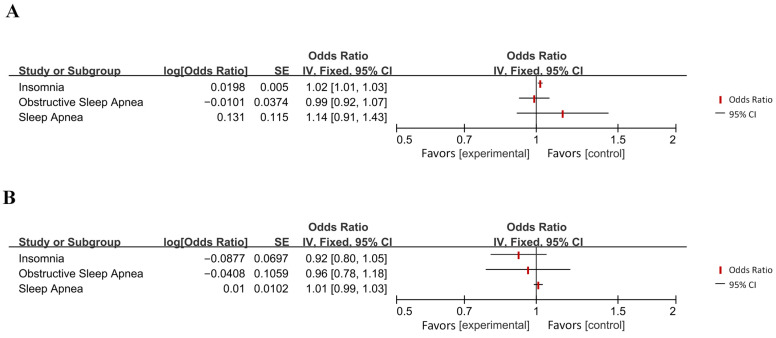
Bidirectional causal association between AD and sleep–wake disorders. (**A**) The results of MR analysis of assessing the causal effects of sleep–wake disorders on AD. (**B**) The results of MR analysis of assessing the causal effects of AD on sleep–wake disorders.

Regarding sleep–wake disorders, insomnia presented a positive association with AD risk (OR = 1.02; 95% CI: 1.01–1.03; *p* = 0.0001) (Figure 2A and Figure A4).

**Figure A5:**
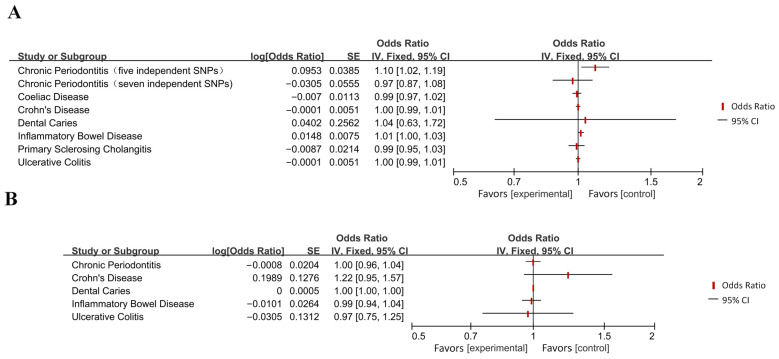
Bidirectional causal association between AD and diseases of the digestive system. (**A**) The results of MR analysis of assessing the causal effects of diseases of the digestive system on AD. (**B**) The results of MR analysis of assessing the causal effects of AD on diseases of the digestive system.

In the realm of digestive system diseases, chronic periodontitis (five SNPs) (OR = 1.10; 95% CI: 1.02–1.19; *p* = 0.013) and inflammatory bowel disease (OR = 1.01; 95% CI: 1.00–1.03; *p* = 0.049) showed genetic correlations with AD (Figure 2A and Figure A5).

**Figure A6:**
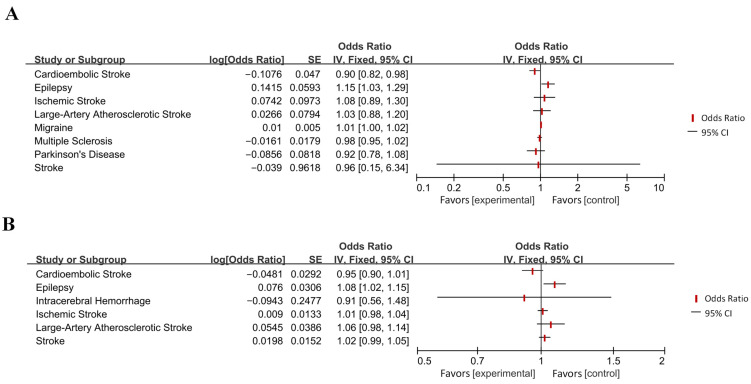
Bidirectional causal association between AD and diseases of the nervous system. (**A**) The results of MR analysis of assessing the causal effects of diseases of the nervous system on AD. (**B**) The results of MR analysis of assessing the causal effects of AD on diseases of the nervous system.

Within nervous system diseases, migraine (OR = 1.01; 95% CI: 1.00–1.02; *p* = 0.046) and epilepsy (OR = 1.15; 95% CI: 1.03–1.29; *p* = 0.017) were positively associated with AD risk, whereas cardioembolic stroke exhibited an inverse association (OR = 0.90; 95% CI: 0.82–0.98; *p* = 0.022) (Figure 2A and Figure A6). Reverse MR analysis indicated that AD genetic liability increased epilepsy risk (OR = 1.08; 95% CI: 1.02~1.15; *p* = 0.013) (Figure 2B and Figure A6).

**Figure A7:**
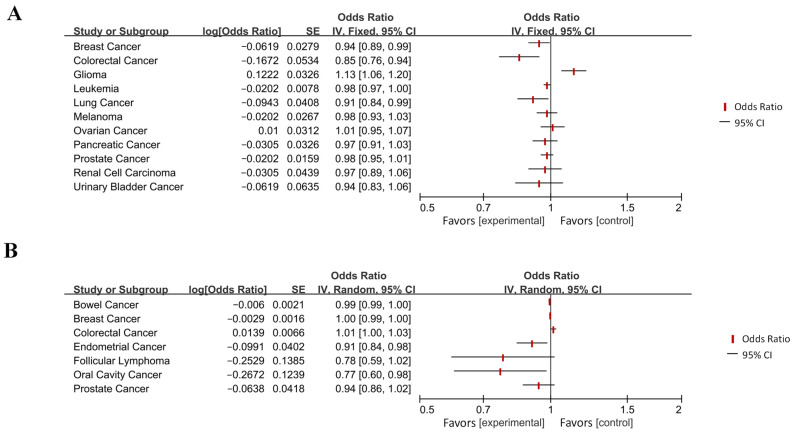
Bidirectional causal association between AD and neoplasms. (**A**) The results of MR analysis of assessing the causal effects of neoplasms on AD. (**B**) The results of MR analysis of assessing the causal effects of AD on neoplasms.

In terms of neoplasms, glioma (OR = 1.13; 95% CI: 1.06–1.20; *p* = 0.0002) was positively associated with AD, while breast cancer (OR = 0.94; 95% CI: 0.89~0.99; *p* = 0.027), colorectal cancer (OR = 0.85; 95% CI: 0.76~0.94; *p* = 0.002), leukemia (OR = 0.98; 95% CI: 0.97~1.00; *p* = 0.010), and lung cancer (OR = 0.91; 95% CI: 0.84~0.99; *p* = 0.021) showed inverse genetic correlations (Figure 2A and Figure A7). Reverse MR revealed causal relationships between AD and oral cancer (OR = 0.77; 95% CI: 0.60~0.98; *p* = 0.031) and endometrial cancer (OR = 0.91; 95% CI: 0.84~0.98; *p* = 0.014). AD genetic risk was further associated with increased colorectal cancer susceptibility (R = 1.01; 95%CI: 1.001~1.027; *p* = 0.035) (Figure 2B and Figure A7).

**Figure A9:**
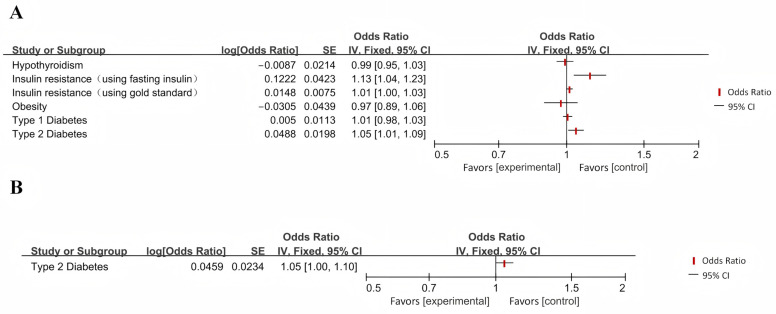
Bidirectional causal association between AD and endocrine, nutritional, or metabolic diseases. (**A**) The results of MR analysis of assessing the causal effects of endocrine, nutritional, or metabolic diseases on AD. (**B**) The results of MR analysis of assessing the causal effects of AD on endocrine, nutritional, or metabolic diseases.

Regarding endocrine, nutritional, or metabolic diseases, type II diabetes (OR = 1.05; 95% CI: 1.01–1.09; *p* = 0.014), insulin resistance (based on the “gold standard” measurement) (OR = 1.01; 95% CI: 1.00–1.03; *p* = 0.049), and insulin resistance (based on fasting insulin) (OR = 1.13; 95% CI: 1.04–1.23; *p* = 0.004) demonstrated positive genetic associations with AD (Figure 2A and Figure A9). Reverse MR analysis showed that AD genetic liability increased type II diabetes risk (OR = 1.05; 95% CI: 1.00~1.10; *p* = 0.0498) (Figure 2B and Figure A9).

**Figure A13:**
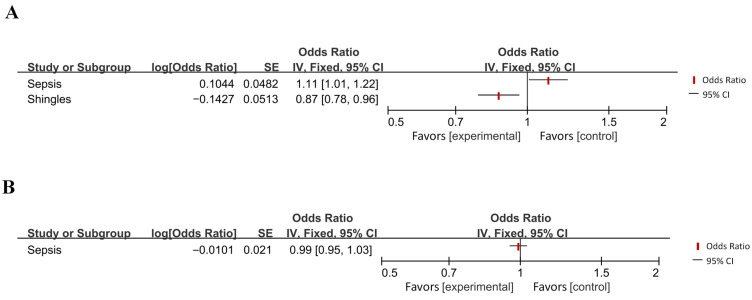
Bidirectional causal association between AD and certain infectious or parasitic diseases. (**A**) The results of MR analysis of assessing the causal effects of certain infectious or parasitic diseases on AD. (**B**) The results of MR analysis of assessing the causal effects of AD on certain infectious or parasitic diseases.

Among infectious or parasitic diseases, analysis shows that sepsis was a genetic predisposition factor that increased AD susceptibility (OR = 1.11; 95% CI: 1.01–1.22; *p* = 0.03), and shingles was inversely associated with AD risk (OR = 0.87; 95% CI: 0.78–0.96; *p* = 0.005) (Figure 2A and Figure A13).

**Figure A14:**
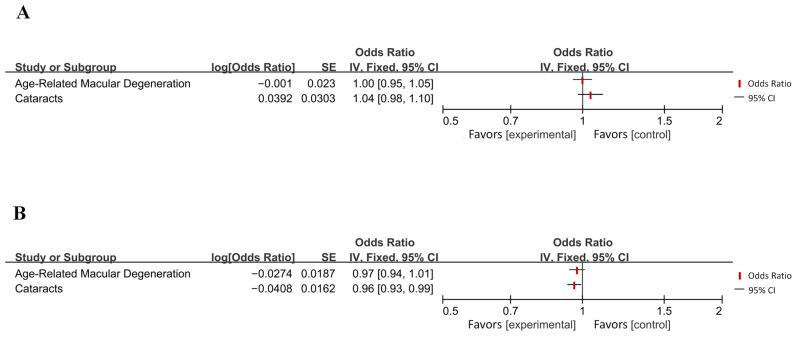
Bidirectional causal association between AD and diseases of the visual system. (**A**) The results of MR analysis of assessing the causal effects of diseases of the visual system on AD. (**B**) The results of MR analysis of assessing the causal effects of AD on diseases of the visual system.

In diseases of the visual system, AD genetic liability was associated with reduced cataract risk (OR = 0.96; 95% CI: 0.93–0.99; *p* = 0.012) (Figure 2B and Figure A14).

## 4. Discussion

Alzheimer’s disease (AD) is a complex neurodegenerative disorder resulting from the interplay of multiple factors. Despite extensive research, no effective treatment for AD has been developed to date. Existing therapies targeting the deficiency of cholinergic neurotransmitters have shown limited success in improving cognitive function. The high failure rate of drug development for AD suggests that treatments focusing on a single factor may not be sufficient. In light of this, the present study conducted a meta-analysis of published Mendelian randomization (MR) studies exploring the relationship between AD and various disease phenotypes. By identifying diseases associated with an increased risk of AD, we aim to propose mechanistic hypotheses for target identification and guide hypothesis-driven therapeutic development, with the ultimate goal of accelerating the discovery of effective AD treatments.

To assess the quality of the included MR studies, we used a modified version of the STROBE-MR guidelines instead of the original [71]. While the STROBE-MR guidelines are widely recognized for reporting MR studies, there is no consensus on tools specifically designed to evaluate bias risk in MR meta-analyses. To fill this gap, we adopted a modified 14-item checklist based on recent systematic reviews [9,10]. This version is more suitable for systematic reviews and meta-analyses, offering additional criteria for evaluating multiple studies and introducing a quantifiable scoring system. This system enhances objectivity, facilitates cross-study comparison, and enables bias risk classification. Moreover, the tool refines the evaluation of bias and methodological consistency, crucial for assessing the quality and reliability of pooled MR results. These adaptations establish a robust, transparent framework, making the modified version a scientifically justified choice for our study.

Ultimately, our findings establish a causal relationship between genetic susceptibility to Alzheimer’s disease (AD) and 10 systemic diseases: musculoskeletal or connective tissue diseases (e.g., hip osteoarthritis, gout); mental, behavioral, or neurodevelopmental disorders (e.g., depression, delirium); circulatory system diseases (e.g., coronary artery disease, hypertension, heart failure, angina); sleep–wake disorders (e.g., insomnia); digestive system diseases (e.g., chronic periodontitis, inflammatory bowel disease); nervous system diseases (e.g., migraine, cardioembolic stroke, ischemic stroke, epilepsy); neoplasms (e.g., glioma, breast cancer, leukemia, lung cancer, oral cancer, endometrial cancer, colorectal cancer); endocrine, nutritional, or metabolic diseases (e.g., type II diabetes, insulin resistance); infectious or parasitic diseases (e.g., shingles); and visual system diseases (e.g., cataracts). These associations suggest links between AD and inflammation, tumors, psycho-behavioral or neurodevelopmental disorders, circulatory system diseases, and endocrine or metabolic disorders.

Inflammation is a manifestation of the immune system’s response, and multiple studies have shown that inflammation may play a very complex role in the progression of Alzheimer’s disease. In recent years, researchers have been dedicated to studying the causal relationship between AS and AD. Previous studies have found that long-term inflammation caused by AS leads to changes in brain connectivity, resulting in brain dysfunction [67]. The commonly used drug TNFi in AS treatment has been found to reduce the risk of AD. In addition, studies have shown that microglia in the brain immune process have both protective and destructive effects on neurons in an inflammatory environment [68]. Apoptosis of both chondrocytes [69] and astrocytes during inflammation [68] activates the TLR3 signaling pathway, producing anti-inflammatory factors and ultimately mediating neuroprotective effects. These mechanisms may partly explain the observed associations between arthritis-related conditions and AD risk.

In individual cancers, lung cancer, breast cancer, and leukemia have been found to have a significant statistical correlation with the reduction in AD risk. At the genetic level, genes related to the increase in cancer risk in any part are all related to a reduction in AD risk [72]. This inverse relationship may stem from the shared dysregulation of key biological pathways between cancer and AD. For instance, tumor suppressor TP53 is downregulated in cancer but upregulated in AD, while PIN1, a cell proliferation promoter, is regulated oppositely in the two conditions [73]. Conversely, increased genetic susceptibility to AD has been linked to a lower incidence of certain cancers. A potential mediator of this relationship may be very-low-density lipoprotein (VLDL), with the immune system also playing a significant role [74]. Immune regulation is likely involved, as AD is characterized by chronic neuroinflammation and cancer by immune evasion, suggesting a complex interplay of immune responses in both diseases [75,76,77]. However, the precise mechanisms underlying this inverse relationship require further experimental and clinical validation.

Genetic susceptibility to mental, behavioral, or neurodevelopmental disorders, such as depression, is associated with an increased risk of Alzheimer’s disease (AD). Conversely, genetic susceptibility to AD appears to elevate the risk of delirium. Several prospective studies have reported a link between depression and an increased risk of dementia. Harerimana et al. [15] identified 75 brain transcripts and 28 proteins with genetic correlations, many of which were associated with AD diagnosis, pathology, or cognitive trajectory. Although Mendelian randomization (MR) studies did not find significant causal relationships between AD and six other mental disorders, potential links remain. The association between ADHD and AD is limited and may be confounded by shared environmental factors. Anxiety disorders [70], ASD [78], BIP [79], and delirium [36] may potentially share pathophysiological mechanisms with AD. Both schizophrenia and AD exhibit structural abnormalities in brain imaging, but current MR studies provide limited evidence for a causal relationship between mental disorders other than depression and AD. Further research is needed to clarify the contribution of mental, behavioral, or neurodevelopmental disorders to AD risk. Regarding reverse causality, the genetic prediction of AD primarily suggests an association with delirium, a multifactorial condition often involving neurotransmission disruptions and neuroinflammation. AD patients are more susceptible to neuroinflammatory cytokines, which may provide a mechanistic link between the two conditions.

Our study identifies a genetic causal relationship between circulatory system diseases and Alzheimer’s disease (AD). Notably, coronary artery disease (CAD) is positively associated with AD risk, potentially linked to elevated apolipoprotein B (APOB) levels. Epidemiological and genetic studies have shown a positive correlation between elevated APOB levels and CAD. Recently, Leah Martin et al. conducted a two-sample Mendelian randomization (MR) analysis, confirming that genetically predicted higher APOB levels increase AD risk [80]. Thus, APOB may explain the observed relationship between CAD and AD in our study. Furthermore, APOB immunoreactivity in senile plaques and neurofibrillary tangles in AD patients supports its role in amyloid deposition and tau pathology [81]. Further genetic correlation analyses have shown a negative association between APOB and healthspan, with AD being a major contributor to reduced healthspan. This reinforces the hypothesis that APOB may influence lifespan through its involvement in AD pathogenesis [80]. Future research focused on regulating APOB may offer promising therapeutic strategies to mitigate the risks associated with both cardiovascular and neurodegenerative diseases.

The causal relationship between insulin resistance and Alzheimer’s disease (AD) may involve multiple mechanisms beyond amyloid-beta accumulation and tau hyperphosphorylation, which are common pathological features of AD. Insulin resistance is linked to an increased risk of atherosclerosis, subclinical inflammation, oxidative stress, and impaired brain perfusion, all of which may contribute to neuronal dysfunction. Zhuang et al. [34] found that middle-aged obesity increases AD risk, while obesity later in life is inversely correlated with AD risk. Although two-sample Mendelian randomization (2SMR) analysis did not reveal a direct causal link, the identification of CNR1 as a potential protective factor for AD offers a promising target for future drug development.

This study has certain limitations. First, its findings may not be generalizable to populations with diverse ethnic or geographic backgrounds, as most included studies focused on individuals of European ancestry, with limited representation from Asian, African, and American populations. Further research is needed to validate these results across broader populations. Secondly, quality assessment using the modified STROBE-MR checklist revealed variability in reporting: 31 studies had a low risk of bias, 23 moderate, and 6 high. While most demonstrated methodological rigor, a substantial portion had limitations that could affect the results’ reliability. Bias risks stemmed from incomplete reporting, methodological inconsistencies, or inadequate justification for instrumental variable selection. Additionally, the potential for horizontal pleiotropy in the original MR studies should be considered. While Mendelian randomization methods mitigate confounding, some studies may not have explicitly tested for pleiotropy. This limitation could introduce bias in the estimated causal associations. Future MR studies should incorporate systematic pleiotropy testing, and future meta-analyses should consider sensitivity analyses that account for pleiotropy when sufficient data are available. Moreover, disease phenotypes were treated as binary variables, without accounting for subtypes or severity, limiting the assessment of their influence. And, the literature classification did not strictly adhere to ICD-11 criteria, and some studies included syndromes overlapping with ICD-11 diseases. Despite careful review, potential misclassification may have occurred during the initial title and abstract screening. Furthermore, some MR studies using non-overlapping GWAS sources may have been omitted from the meta-analysis. Additionally, no restrictions were placed on the number of SNPs used, and studies with fewer than 10 genetic instruments were included, potentially introducing variability. As MR research advances, integrating methods like multivariable and mediation analysis will help account for confounders such as medication use, smoking, and lifestyle, enhancing causal inference accuracy.

Despite these limitations, our findings suggest that systemic diseases, particularly those related to inflammation, neurodevelopmental disorders, and metabolic conditions, may influence AD risk, offering new avenues for prevention and treatment. The inverse relationship between AD and certain cancers also presents potential therapeutic opportunities. Future research should focus on further elucidating the mechanisms underlying these complex interactions. Prospective cohort studies with diverse populations are needed to validate these causal relationships and examine the effects of disease subtypes and severity on AD risk. Policy efforts should prioritize integrating genetic risk factors into early AD diagnosis and treatment strategies, ensuring broader demographic inclusivity in research.

## Figures and Tables

**Table 1:** Characteristics of studies included in meta-analysis.

Study (First Author and Year of Publication)	Disease	Number of Disease Cases	Sample Size	Population
Bae et al. (2019) [12]	Rheumatoid arthritis	29,880	103,638	European
Cai et al. (2021) [13]	Osteoarthritis	77,052	455,211	European
Lee et al. (2019) [14]	Gout	968, 4275, 945	16,474, 10,547, 2158	European, Chinese, Japanese
Harerimana et al. (2022) [15]	Depression	246,363	807,553	European
Pagoni et al. (2022) [16]	Hyperactivity	20,183	55,374	European
	Autism spectrum disorder	18,381	46,350	European
Wei et al. (2022) [17]	Anxiety disorder	25,453	83,566	European
	Bidirectional affective disorder	20,352	51,710	European
	Schizophrenia	33,640	77,096	European
Zheng et al. (2023) [18]	Delirium	2612	327,918	European
Zhang et al. (2023) [19]	Coronary heart disease	22,233	86,995	European
	Myocardial infarction	11,622	199,462	European
	Atrial fibrillation	65,446	588,190	European
	Angina pectoris	18,168	206,520	European
	Ischemic stroke	34,217	440,328	European
	Large atherosclerotic stroke	4373	410,484	European
	Cardioembolic stroke	7193	413,304	European
Arega et al. (2022) [20]	Heart failure	47,309	977,323	European
Grace et al. (2018) [21]	Coronary artery disease	60,801	184,305	European
Tang et al. (2023) [22]	Hypertension	/	/	European
Chen et al. (2022) [23]	Insomnia	109,402	386,533	European
Li et al. (2022) [24]	Obstructive sleep disorder	16,761	217,955	European
Cavaillès et al. (2023) [25]	Sleep apnea	20,008	523,366	European
Enduru et al. (2024) [26]	Celiac disease	4533	15,283	European
	Crohn’s disease	12,194	40,266	European
	Primary sclerosing cholangitis	4796	309,154	European
	Ulcerative colitis	12,366	45,975	European
	Specific dermatitis	10,788	40,835	European
	Vitiligo	4680	44,266	European
	Hypothyroidism	13,043	244,890	European
	Asthma	44,301	385,822	European
	Allergic rhinitis	22,057	289,307	European
	Systemic lupus erythematosus	7219	23,210	European
	Rheumatoid arthritis	14,361	58,284	European
Liao et al. (2023) [27]	Dental caries	/	/	European
Zeng et al. (2023) [28]	Inflammatory bowel disease	25,042	About 60,000	European
Zeng et al. (2023) [29]	Inflammatory bowel disease	3753	219,559	European
Sun et al. (2020) [30]	Chronic periodontitis	4924, 12,289	12,225, 34,615	European
Shen et al. (2023) [31]	Bullous pemphigoid	282	218,348	European
Zhou et al. (2022) [32]	Insulin resistance	/	/	European
Xue et al. (2023) [33]	Type I diabetes	9266	24,840	European
	Multiple sclerosis	47,429	115,803	European
	Migraine	102,084	873,341	European
Zhuang et al. (2021) [34]	Obesity	32,858	98,697	European
Han et al. (2018) [35]	Parkinson’s disease	26,035	428,235	European
Xu et al. (2023) [36]	Epilepsy	803	30,480	European
Wang et al. (2020) [37]	Stroke	67,162	521,612	European
Wu et al. (2021) [38]	Glioma	/	/	European
Seddighi et al. (2019) [39]	Breast cancer	/	/	European
	Lung cancer	/	/	European
	Leukemia	/	/	European
Abidin et al. (2021) [40]	Hearing difficulties	87,056	250,389	European
Huang et al. (2021) [41]	Zoster	16,711	134,963	European
Jiang et al. (2022) [42]	Age-related macular degeneration	16,144	33,976	European
Man et al. (2023) [43]	Cataracts	67,844	585,243	European
Liu et al. (2023) [44]	Chronic kidney disease	/	/	European
Sheng et al. (2022) [45]	Gestational diabetes	2062	/	European
Grover et al. (2022) [46]	Insomnia	109,389	386,533	European
Tang (2022) [47]	Asthma	23,948	142,486	European
Fu et al. (2022) [48]	Gout	/	/	European
Ou et al. (2022) [49]	Gout	2115	69,374	European
Li et al. (2022) [50]	Crohn’s disease	5956	20,883	European
	Ulcerative colitis	6968	27,432	European
Pan et al. (2020) [51]	Atrial fibrillation	60,620	1,030,836	European
Daghlas et al. (2020) [52]	Migraine	59,674	375,752	European
Zhou et al. (2023) [53]	Ankylosing spondylitis	9069	22,647	European
Cai et al. (2018) [54]	Rheumatoid arthritis	14,361	58,284	European
Cui et al. (2022) [55]	Inflammatory bowel disease	25,042	59,957	European
	Ulcerative colitis	12,366	45,975	European
	Crohn’s disease	12,194	40,266	European
Dong et al. (2023) [56]	Endometrial cancer, etc.	/	/	European
Duan et al. (2021) [57]	Heart failure	47,309	977,323	European
Li et al. (2022) [58]	Apoplexy	40,585	446,696	European
	Cerebral hemorrhage	1545	3026	European
	Ischemic stroke	34,217	440,328	European
Tang et al. (2023) [59]	Osteoporosis	3203	212,778	European
Liu et al. (2022) [60]	Obstructive sleep apnea	20,279	259,404	European
Zhang et al. (2020) [61]	Type II diabetes	/	659,316	European
Dybjer et al. (2023) [62]	Type II diabetes	74,124	898,130	European
Litkowski et al. (2023) [63]	Type II diabetes	82,980	334,672	European
Luo et al. (2023) [64]	Type II diabetes	39,106	440,683	European
Meng et al. (2022) [6]	Type II diabetes	48,286	298,957	European
Pan et al. (2020) [65]	Type II diabetes	/	212,747	European
Gao et al. (2023) [66]	Atrial fibrillation	/	/	/
Kuo et al. (2022) [67]	Heart failure	/	/	/
Wang et al. (2023) [68]	Hip osteoarthritis	/	/	/
	Ankylosing spondylitis	/	/	/
	Rheumatoid arthritis	/	/	/
Yuan et al. (2024) [69]	Colorectal cancer	/	/	/
Zeng et al. (2024) [70]	Sepsis	/	/	/

**Table 2:** Quality assessment of all included studies.

Study (First Author and Year of Publication)	Items *														Total Score (Out of 14)	%
	Title and Abstract		Rationale and Objectives		Study Design			Reporting		Analysis		Assessment of Assumptions				
	1	2	3	4	5	6	7	8	9	10	11	12	13	14		
Bae et al. (2019) [12]	+	+	+	·	+		+	+	+	+	+	+	+	·	12.5	89.3
Cai et al. (2021) [13]	+	+	+	·	+	·	+	+	+	+	+	+	+	+	13	92.9
Lee et al. (2019) [14]	+	+	+	·	+	·	+	+	+	+	+	·	+	+	12.5	89.3
Harerimana et al. (2022) [15]	+	+	+	+	+	−	+	+	+	+	+	+	+	·	12.5	89.3
Pagoni et al. (2022) [16]	+	+	+	+	+	·	·	·	+	+	+	+	+	+	12.5	89.3
Wei et al. (2022) [17]	+	+	+	+	+	·	+	+	+	+	+	+	+	·	13	92.9
Zheng et al. (2023) [18]	+	+	+	·	+	·	+	+	+	+	+	+	+	+	13	92.9
Zhang et al. (2023) [19]	+	+	+	+	+	·	+	+	+	+	+	+	+	+	13.5	96.4
Arega et al. (2022) [20]	+	+	+	·	+	·	+	+	+	+	+	+	+	+	13	92.9
Grace et al. (2018) [21]	+	+	+	+	+	·	+	+	+	+	+	+	+	−	12.5	89.3
Tang et al. (2023) [22]	+	+	+	·	+	+	·	+	+	+	+	+	+	+	13	92.9
Chen et al. (2022) [23]	+	−	+	+	+	·	+	·	+	+	+	+	+	−	12	85.7
Li et al. (2022) [24]	+	+	+	+	+	·	+	+	+	+	+	+	+	+	13.5	96.4
Cavaillès et al. (2023) [25]	+	+	+	+	+	·	+	+	+	+	+	+	+	+	13.5	96.4
Enduru et al. (2024) [26]	−	+	+	·	+	·	+	·	+	+	+	+	+	·	11	78.6
Liao et al. (2023) [27]	+	+	+	+	+	·	+	+	+	+	+	+	+	+	13.5	96.4
Zeng et al. (2023) [28]	+	+	+	·	+	·	+	+	+	+	+	+	+	+	13	92.9
Zeng et al. (2023) [29]	+	+	+	·	+	·	+	+	·	+	+	+	·	−	11	78.6
Sun et al. (2020) [30]	+	+	+	+	+	·	+	+	·	+	+	·	+	+	12.5	89.3
Shen et al. (2023) [31]	+	+	+	·	+	·	+	+	+	+	+	+	+	+	13	92.9
Zhou et al. (2022) [32]	+	+	+	+	+	·	−	+	+	+	+	+	+	+	12.5	89.3
Xue et al. (2023) [33]	+	+	+	·	+	·	+	+	+	+	+	+	+	+	13	92.9
Zhuang et al. (2021) [34]	+	+	+	+	+	·	+	+	·	+	+	+	·	−	11.5	82.1
Han et al. (2018) [35]	+	+	+	·	+	·	+	+	+	+	+	+	·	+	12.5	89.3
Xu et al. (2023) [36]	+	+	+	+	+	·	+	+	+	+	+	+	+	+	13.5	96.4
Wang et al. (2020) [37]	+	+	+	+	+	·	+	+	+	+	+	+	·	+	13	92.9
Wu et al. (2021) [38]	+	·	+	−	+	·	+	+	+	+	+	·	+	+	11.5	82.1
Seddighi et al. (2019) [39]	+	+	+	·	+	·	+	+	+	+	+	+	+	+	13	92.9
Abidin et al. (2021) [40]	·	+	+	+	+	·	+	+	+	+	·	+	−	+	11.5	82.1
Huang et al. (2021) [41]	+	+	+	+	+	·	+	+	+	+	+	+	+	·	13	92.9
Jiang et al. (2022) [42]	+	+	+	+	+	·	+	+	+	+	+	+	+	+	13.5	96.4
Man et al. (2023) [43]	+	+	+	·	+	·	+	+	+	+	+	+	+	+	13	92.9
Liu et al. (2023) [44]	+	+	+	·	+	·	+	+	+	+	+	+	+	+	13	92.9
Sheng et al. (2022) [45]	+	+	+	+	+	·	+	+	+	+	+	+	+	+	13.5	96.4
Grover et al. (2022) [46]	+	+	+	·	+	·	+	+	+	+	+	+	−	−	11	78.6
Tang et al. (2022) [47]	+	+	+	·	+	·	·	·	·	+	+	+	+	+	11.5	82.1
Fu et al. (2022) [48]	−	+	·	+	+	·	+	+	+	+	+	+	+	−	11	78.6
Ou et al. (2022) [49]	+	+	+	+	+	·	+	+	+	+	+	+	+	+	13.5	96.4
Li et al. (2022) [50]	+	+	+	·	+	−	+	+	+	+	+	+	+	+	12.5	89.3
Pan et al. (2020) [51]	+	+	+	+	+	·	+	+	+	+	+	+	+	−	12.5	89.3
Daghlas et al. (2020) [52]	+	+	+	+	+	·	+	+	·	+	+	+	+	−	12	85.7
Zhou et al. (2023) [53]	+	+	+	·	+	·	+	+	+	+	+	+	+	+	13	92.9
Cai et al. (2018) [54]	+	+	+	·	+	·	+	+	+	+	−	·	·	+	11	78.6
Cui et al. (2022) [55]	+	+	+	·	+	·	+	+	+	+	+	+	+	+	13	92.9
Dong et al. (2023) [56]	+	+	+	·	+	·	+	·	+	+	+	+	−	+	11.5	82.1
Duan et al. (2021) [57]	+	+	+	+	+	·	+	+	+	+	·	+	+	+	13	92.9
Li et al. (2022) [58]	+	+	+	+	+	·	+	+	+	+	+	+	+	−	12.5	89.3
Tang et al. (2023) [59]	+	+	+	·	+	·	+	+	+	+	+	+	+	−	12	85.7
Liu et al. (2022) [60]	+	+	+	+	+	·	+	+	·	+	+	+	+	−	12	85.7
Zhang et al. (2020) [61]	+	+	+	−	+	·	−	+	+	+	+	·	+	−	10	71.4
Dybjer et al. (2023) [62]	+	+	+	+	+	·	+	+	+	+	+	+	+	−	12.5	89.3
Litkowski et al. (2023) [63]	+	+	+	+	+	·	+	+	+	+	+	+	+	+	13.5	96.4
Luo et al. (2023) [64]	·	−	+	+	+	·	+	+	+	+	+	+	+	·	11.5	82.1
Meng et al. (2022) [6]	+	+	+	·	+	·	+	+	+	+	+	+	+	−	12	85.7
Pan et al. (2020) [65]	+	+	+	+	+	·	+	+	+	+	+	+	+	+	13.5	96.4
Gao et al. (2023) [66]	+	+	+	+	+	.	+	+	+	+	+	+	+	+	13.5	96.4
Kuo et al. (2022) [67]	+	+	+	+	+	+	+	+	+	+	+	+	+	+	14	100
Wang et al. (2023) [68]	+	+	+	.	+	.	+	+	+	+	+	+	+	+	13	92.9
Yuan et al. (2024) [69]	+	+	+	.	+	.	+	+	+	+	+	+	+	+	13	92.9
Zeng et al. (2024) [70]	+	+	+	.	+	.	+	+	+	+	+	+	+	+	13	92.9

* For items 1~14, the assessment criteria are as follows: title and abstract, background, objectives, study design and data sources, main statistical methods of analysis, software and pre-registration, descriptive data, main results, sensitivity and additional analyses, main results, limitations, interpretation, generalizability, and Mendelian randomization core assumptions. In the risk-of-bias table, “+” indicates that the item meets the guideline definition and is assigned 1 point; “.” indicates a slight deviation from the guideline definition and is assigned 0.5 points; “−” indicates a significant deviation from the guideline definition and is assigned 0 points.

**Table 3:** Summary of bidirectional causal effects between Alzheimer’s disease and various systemic diseases (significant results section).

Disease Category	Specific Disease	OR [95% CI]	*p*-Value ^1^	Effect ^2^	Directionality ^3^
Diseases of the musculoskeletal system or connective tissue	Gout	1.02 [1.00, 1.05]	0.049 (*)	Increased Risk	→ AD
	Hip osteoarthritis	0.80 [0.69, 0.94]	0.007 (**)	Reduced Risk	→ AD
	Rheumatoid arthritis	0.97 [0.95, 0.99]	0.004 (**)	Reduced Risk	→ AD
Mental, behavioral, or neurodevelopmental disorders	Depression	1.03 [1.01, 1.05]	0.001 (**)	Increased Risk	→ AD
	Delirium	1.32 [1.13, 1.54]	0.0005 (***)	Increased Risk	AD →
Diseases of the circulatory system	Coronary artery disease	1.07 [1.01, 1.13]	0.021 (*)	Increased Risk	→ AD
	Hypertension	4.30 [1.04, 17.78]	0.044 (*)	Increased Risk	→ AD
	Angina	1.06 [1.02, 1.10]	0.003 (**)	Increased Risk	AD →
Sleep–wake disorders	Insomnia	1.02 [1.01, 1.03]	0.0001 (***)	Increased Risk	→ AD
Diseases of the digestive system	Chronic periodontitis (5 independent SNPs)	1.10 [1.02, 1.19]	0.013 (*)	Increased Risk	→ AD
	Inflammatory bowel disease	1.01 [1.00, 1.03]	0.049 (*)	Increased Risk	→ AD
Endocrine, nutritional, or metabolic disease	Insulin resistance (using gold standard)	1.01 [1.00, 1.03]	0.049 (*)	Increased Risk	→ AD
	Insulin resistance (using fasting insulin)	1.13 [1.04, 1.23]	0.004 (**)	Increased Risk	→ AD
	Type II diabetes	1.05 [1.01, 1.09]/1.05 [1.00, 1.10]	0.014 (*)/0.0498 (*)	Increased Risk/Increased Risk	↔
Diseases of the nervous system	Cardioembolic stroke	0.90 [0.82, 0.98]	0.022 (*)	Reduced Risk	→ AD
	Epilepsy	1.15 [1.03, 1.29]/1.08 [1.02, 1.15]	0.017 (*)/0.013 (*)	Increased Risk/Increased Risk	↔
	Migraine	1.01 [1.00, 1.02]	0.046 (*)	Increased Risk	→ AD
Certain infectious or parasitic diseases	Sepsis	1.11 [1.01, 1.22]	0.03 (*)	Reduced Risk	AD →
	Shingles	0.87 [0.78, 0.96]	0.005 (**)	Reduced Risk	→ AD
Neoplasms	Breast cancer	0.94 [0.89, 0.99]	0.027 (*)	Reduced Risk	→ AD
	Colorectal cancer	0.85 [0.76, 0.94]/1.01 [1.001, 1.027]	0.002 (**)/0.035 (*)	Reduced Risk/Increased Risk	↔
	Glioma	1.13 [1.06, 1.20]	0.0002 (***)	Increased Risk	→ AD
	Leukemia	0.98 [0.97, 1.00]	0.010 (**)	Reduced Risk	→ AD
	Lung cancer	0.91 [0.84, 0.99]	0.021 (*)	Reduced Risk	→ AD
	Bowel cancer	0.99 [0.99, 1.00]	0.004(**)	Reduced Risk	AD →
	Endometrial cancer	0.91 [0.84, 0.98]	0.014 (*)	Reduced Risk	AD →
	Oral cancer	0.77 [0.60, 0.98]	0.031 (*)	Reduced Risk	AD →
Diseases of the visual system	Cataracts	0.96 [0.93, 0.99]	0.012 (*)	Reduced Risk	AD →

^1^ (*) *p* < 0.05, (**) *p* < 0.01, and (***) *p* < 0.001. ^2^ Reduced Risk: OR < 1 (genetic predisposition associated with lower risk); Increased Risk: OR > 1 (genetic predisposition associated with higher risk). ^3^ → AD: the disease has a genetically predicted causal effect on AD (increased/reduced risk); AD →: AD has a genetically predicted causal effect on the disease (increased/reduced risk); ↔: bidirectional genetically predicted causal effects between AD and the disease. Associations derived from Mendelian randomization studies, indicating that carrying genetic variants predisposing the carrier to the disease is statistically linked to altered AD risk. These findings do not imply that clinically diagnosed diseases directly cause or prevent AD in individuals.

**Figure A8:**
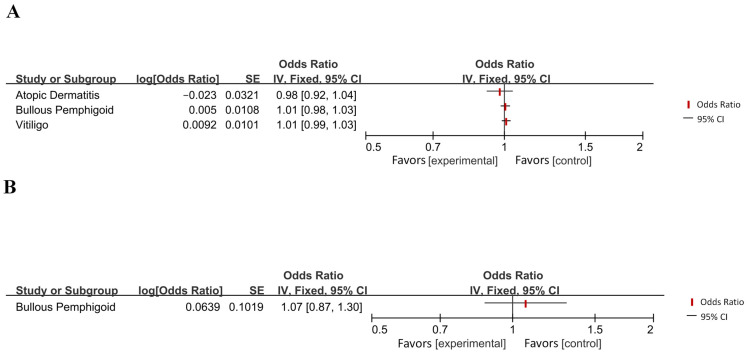
Bidirectional causal association between AD and diseases of the skin. (**A**) The results of MR analysis of assessing the causal effects of diseases of the skin on AD. (**B**) The results of MR analysis of assessing the causal effects of AD on diseases of the skin.

**Figure A10:**
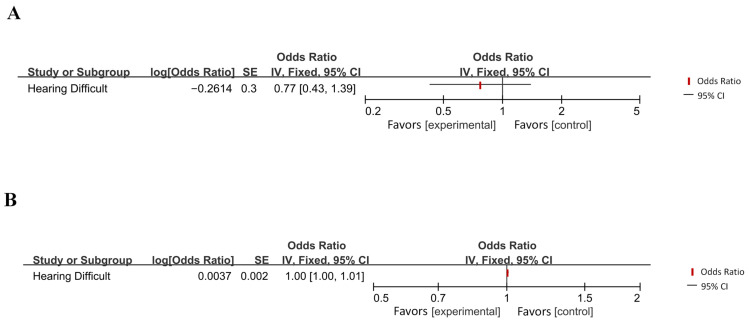
Bidirectional causal association between AD and diseases of the ear or mastoid process. (**A**) The results of MR analysis of assessing the causal effects of diseases of the ear or mastoid process on AD. (**B**) The results of MR analysis of assessing the causal effects of AD on diseases of the ear or mastoid process.

**Figure A11:**
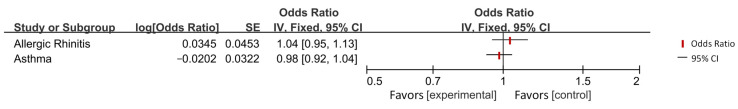
Results of MR analysis of assessing the causal effects of diseases of the respiratory system on AD.

**Figure A12:**
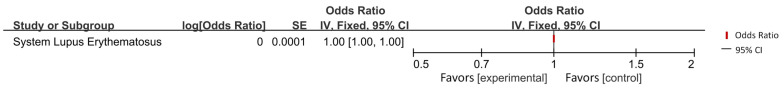
Results of MR analysis of assessing the causal effects of diseases of the immune system on AD.

**Figure A15:**
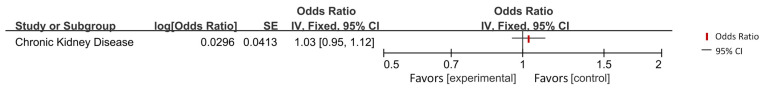
Results of MR analysis of assessing the causal effects of diseases of the genitourinary system on AD.

**Figure A16:**
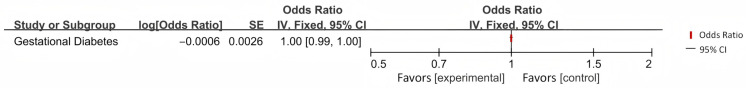
Results of MR analysis of assessing the causal effects of pregnancy, childbirth, or the puerperium on AD.

## Data Availability

The original contributions presented in this study are included in the article. Further inquiries can be directed to the corresponding authors.

## Abbreviations

The following abbreviations are used in this manuscript:

MR	Mendelian randomization
AD	Alzheimer’s disease
PubMed	Public from Medline
CNKI	China National Knowledge Infrastructure
VIP Database	Chinese Science and Technology Periodical Database

## Appendix B

**Table A1:** The retrieval strategy and results.

Database	Search Strategy *
PubMed	(((Mendelian Randomization [Title/Abstract]) OR (Mendelian Randomization Analysis [Title/Abstract])) OR (Mendelian Randomization Study [Title/Abstract])) OR (Mendelian Randomization Methods [Title/Abstract]) AND ((((((((((((((((((((Alzheimer’s disease [Title/Abstract]) OR (Alzheimer Dementia [Title/Abstract])) OR (Alzheimer Dementias [Title/Abstract])) OR (Dementia, Alzheimer [Title/Abstract])) OR (Alzheimer’s Disease [Title/Abstract])) OR (Dementia, Senile [Title/Abstract])) OR (Dementia, Alzheimer Type [Title/Abstract])) OR (Alzheimer Type Dementia (ATD [Title/Abstract]))) OR (Dementia, Alzheimer-Type (ATD [Title/Abstract]))) OR (Alzheimer Type Senile Dementia [Title/Abstract])) OR (Dementia, Primary Senile Degenerative [Title/Abstract])) OR (Sclerosis, Alzheimer [Title/Abstract])) OR (Alzheimer Syndrome [Title/Abstract])) OR (Alzheimer’s Diseases [Title/Abstract])) OR (Alzheimer Diseases [Title/Abstract])) OR (Alzheimers Diseases [Title/Abstract])) OR (Senile Dementia, Alzheimer Type [Title/Abstract])) OR (Senile Dementia, Acute Confusional [Title/Abstract])) OR (Dementia, Presenile [Title/Abstract])) OR (Alzheimer Disease, Late Onset [Title/Abstract]))
Embase	(Mendelian Randomization or Mendelian Randomization Analysis or Mendelian Randomization Study or Mendelian Randomization Methods).ab. and (alzheimer’s disease or Alzheimer Dementia or Alzheimer Dementias or Dementia, Alzheimer or Alzheimer Syndrome or Alzheimer-Type Dementia or Alzheimer Type Dementia or Dementia, Alzheimer-Type or Alzheimer’s Diseases or Alzheimer Diseases or Alzheimers Diseases or Dementia, Senile or Senile Dementia or Dementia, Alzheimer Type or Alzheimer Type Dementia or Senile Dementia, Alzheimer Type or Alzheimer Type Senile Dementia or Primary Senile Degenerative Dementia or Dementia, Primary Senile Degenerative or Sclerosis, Alzheimer or Senile Dementia, Acute Confusional).ab.
CNKI	TKA = (‘阿尔茨海默病’ + ‘阿尔兹海默’ + ‘阿尔茨海默’ + ‘阿尔兹海默病’) AND TKA = (‘孟德尔随机化’ + ‘孟德尔随机化方法’ + ‘孟德尔随机化法’ + ‘孟德尔随机化研究’ + ‘孟德尔随机化试验’ + ‘孟德尔随机化分析’ + ‘孟德尔随机分析’ + ‘孟德尔随机方法’)
Wanfang Data	摘要:(阿尔兹海默症 or 阿尔兹海默 or 阿尔茨海默病) and 摘要:(孟德尔随机化 or 孟德尔随机化方法 or 孟德尔随机化法 or 孟德尔随机化研究 or 孟德尔随机化试验 or 孟德尔随机化分析 or 孟德尔随机分析 or 孟德尔随机方法)
VIP Database	U = (阿尔兹海默症 OR 阿尔兹海默 OR 阿尔茨海默病) AND (孟德尔随机化 OR 孟德尔随机化方法 OR 孟德尔随机化法 OR 孟德尔随机化研究 OR 孟德尔随机化试验 OR 孟德尔随机化分析 OR 孟德尔随机分析 OR 孟德尔随机方法)

* The search terms used in Chinese-language databases (CNKI, Wanfang Data, VIP Database) include variations of “Alzheimer’s disease” (阿尔茨海默病, 阿尔兹海默症, 阿尔兹海默) and “Mendelian randomization” (孟德尔随机化, 孟德尔随机化方法, 孟德尔随机化法, 孟德尔随机化研究, 孟德尔随机化试验, 孟德尔随机化分析, 孟德尔随机分析, 孟德尔随机方法). These terms are tailored to the Chinese-language search requirements, ensuring the accuracy and relevance of the results.

**Table A2:** Extracted data from included studies for meta-analysis.

Study (First Author and Year of Publication)	Exposure	Outcome	Method	OR	95%CI	*p*-Value
Bae et al. (2019) [12]	RA	AD	IVW	0.96	0.93–0.99	0.021
Cai et al. (2021) [13]	Osteoarthritis	AD	IVW	1.064	0.928–1.219	0.374
	AD	Osteoarthritis	IVW	0.979	0.972–0.987	1.190 × 10^−7^
Lee et al. (2019) [14]	Gout	AD	IVW	/	/	0.445
Harerimana et al. (2022) [15]	Depression	AD	Forward Generalized Summary data-based Mendelian Randomization (GSMR)	/	/	0.001
	AD	Depression	Reverse Generalized Summary data-based Mendelian Randomization (GSMR)	/	/	0.954
Pagoni et al. (2022) [16]	Autism spectrum disorder	AD	IVW	0.99	0.97–1.01	0.7
	AD	Autism spectrum disorder	IVW	1.19	0.94–1.51	0.14
	Hyperactivity	AD	IVW	1	0.98–1.02	0.39
	AD	Hyperactivity	IVW	1.12	0.86–1.44	0.37
Wei et al. (2022) [17]	Anxiety disorders	AD	IVW	0.904	0.722–1.131	0.377
	Bidirectional affective disorder	AD	IVW	1.033	0.925–1.153	0.564
	Schizophrenia	AD	IVW	1.039	0.986–1.095	0.156
Zheng et al. (2023) [18]	Delirium	AD	IVW	0.98	0.91–1.05	0.544
	AD	Delirium	IVW	1.32	1.13–1.54	0.000391
Zhang et al. (2023) [19]	Coronary heart disease	AD	IVW	0.983	0.905–1.068	0.691
	AD	Coronary heart disease	IVW	0.912	0.802–1.037	0.159
	Myocardial infarction	AD	IVW	1.3	0.791–2.137	0.3
	AD	Myocardial infarction	IVW	1.049	0.993–1.108	0.088
	Atrial fibrillation	AD	IVW	0.994	0.951–1.039	0.78
	AD	Atrial fibrillation	IVW	0.994	0.972–1.017	0.612
	Ischemic stroke	AD	IVW	1.077	0.890–1.304	0.447
	AD	Ischemic stroke	IVW	1.009	0.983–1.036	0.502
	Angina pectoris	AD	IVW	1.117	0.874–1.428	0.375
	AD	Angina pectoris	IVW	1.06	1.020–1.101	0.003
	Cardioembolic stroke	AD	IVW	0.898	0.819–0.986	0.024
	AD	Cardioembolic stroke	IVW	0.953	0.900–1.010	0.104
	Cardioembolic stroke	AD	IVW	1.027	0.879–1.200	0.736
	AD	Cardioembolic stroke	IVW	1.056	0.979–1.139	0.155
Arega et al. (2022) [20]	Heart failure	AD	IVW	0.752	0.621, 0.912	0.004
Grace et al. (2018) [21]	Coronary artery disease	LOAD	IVW	1.07	1.01,1.15	0.027
Tang et al. (2023) [22]	Hypertension	AD	IVW	4.3	1.04–17.6	0.04
Chen et al. (2022) [23]	Insomnia	AD	IVW	1.02		6.7 × 10^−6^
Li et al. (2022) [24]	Obstructive sleep disorder	AD	IVW	0.99	0.92–1.08	0.26
Cavaillès et al. (2023) [25]	Sleep apnea	AD	Fixed-effects IVW	1.14	0.91–1.43	/
	AD	Sleep apnea	Fixed-effects IVW	1.01	0.99–1.02	/
Enduru et al. (2024) [26]	Celiac disease	AD	IVW	/	/	/
	Crohn’s disease	AD	IVW	/	/	/
	Primary sclerosing cholangitis	AD	IVW	/	/	/
	Ulcerative colitis	AD	IVW	/	/	/
	Specific dermatitis	AD	IVW	/	/	/
	Vitiligo	AD	IVW	/	/	/
	Hypothyroidism	AD	IVW	/	/	/
	Asthma	AD	IVW	/	/	/
	Allergic rhinitis	AD	IVW	/	/	/
	Systemic lupus erythematosus	AD	IVW	/	/	/
	Rheumatoid arthritis	AD	IVW	/	/	/
Liao et al. (2023) [27]	Dental caries	AD	IVW	1.041	0.630–0.722	0.874
	AD	Dental caries	IVW	1	0.999–1.001	0.717
Zeng et al. (2023) [28]	Inflammatory bowel disease	AD	IVW	1.01	0.99–1.02	0.41
	AD	Inflammatory bowel disease	IVW	0.99	0.94–1.05	>0.05
Zeng et al. (2023) [29]	Inflammatory bowel disease	AD	IVW	1.015	0.993–1.038	0.179
	AD	Inflammatory bowel disease	IVW	0.979	0.899–1.066	0.628
Sun et al. (2020) [30]	Chronic periodontitis	AD	IVW	1.1; 0.97	1.02–1.19; 0.87–1.08	0.02; 0.59
	AD	Chronic periodontitis	IVW	1	0.96–1.04	0.85
Shen et al. (2023) [31]	Bullous pemphigoid	AD	IVW	1.005	0.984–1.027	0.629
	AD	Bullous pemphigoid	IVW	1.066	0.873–1.358	0.603
Zhou et al. (2022) [32]	Insulin resistance	AD	IVW	1.13	1.04–1.23	0.004
Xue et al. (2023) [33]	Type I diabetes	AD	IVW	1.005	0.983–1.026	0.641
	Multiple sclerosis	AD	IVW	0.984	0.950–1.019	0.381
	Migraine	AD	IVW	20.264	0.124–3287.978	0.246
Zhuang et al. (2021) [34]	Obesity	AD	IVW	0.97	0.89–1.06	0.49
Han et al. (2018) [35]	Parkinson’s disease	AD	IVW	0.918	0.782–1.076	0.291
Xu et al. (2023) [36]	AD	Epilepsy	IVW	1.152	/	0.017
	Epilepsy	AD	IVW	/	/	/
Wang et al. (2020) [37]	Stroke	AD	IVW	/	/	/
Wu et al. (2021) [38]	Glioma	AD	IVW	1.13	1.06–1.21	4.8 × 10^−4^
Seddighi et al. (2019) [39]	Breast cancer	AD	IVW	0.91	0.84–0.99	0.019
	Lung cancer	AD	IVW	0.98	0.96–0.995	0.012
	Leukemia	AD	IVW	0.94	0.89–0.99	0.028
Abidin et al. (2021) [40]	Hearing difficulties	Late-onset AD	IVW	0.770		0.390
	Late-onset AD	Hearing difficulties	IVW	1.004		0.061
Huang et al. (2021) [41]	Zoster	AD	IVW	0.867	0.784–0.958	0.005
Jiang et al. (2022) [42]	Age-related macular degeneration	AD	IVW	0.999	0.955–1.044	0.948
	AD	Age-related macular degeneration	IVW	0.973	0.938–1.008	0.133
Man et al. (2023) [43]	Cataracts	AD	IVW	1.04	0.98–1.10	0.199
	AD	Cataracts	IVW	0.96	0.93–0.99	/
Liu et al. (2023) [44]	Chronic kidney disease	AD	IVW	1.03	0.95–1.12	0.41
Sheng et al. (2022) [45]	Gestational diabetes	AD	IVW	/	/	0.82
Grover et al. (2022) [46]	Insomnia	AD	IVW	1.135	0.826–1.560	0.4017
	AD	Insomnia	IVW	0.916	0.799–1.051	0.2011
Tang (2022) [47]	Asthma	AD	IVW	1.129	0.842–1.515	0.679
Fu et al. (2022) [48]	Gout	AD	IVW	/	/	/
Ou et al. (2022) [49]	Gout	AD	IVW	1.05	1.00–1.11	0.057
	AD	Gout	IVW	0.25	0.67–1.29	0.653
Li et al. (2022) [50]	Crohn’s disease	AD	IVW	1	0.99–1.00	0.26
	AD	Crohn’s disease	IVW	1.2	0.80–1.82	0.38
	Ulcerative colitis	AD	IVW	1	0.99–1.00	0.57
	AD	Ulcerative colitis	IVW	1.14	0.81–1.60	0.44
Pan et al. (2020) [51]	Atrial fibrillation	AD	IVW	1.002	0.996–1.009	0.47
Daghlas et al. (2020) [52]	Migraine	AD	IVW of the variant–outcome association with intercept constrained to zero	1.01	1.00–1.02	0.07
Zhou et al. (2023) [53]	Ankylosing spondylitis	AD	IVW	0.976	0.955–0.997	0.029
Cai et al. (2018) [54]	AD	Rheumatoid arthritis	IVWweighted medianMR Egger	0.950.96, 0.98	0.88–1.03; 0.85–1.07; 0.78–1.22	0.451; 0.217; 0.827
Cui et al. (2022) [55]	AD	Inflammatory bowel disease	IVW	/	0.736–1.331	0.945
	AD	Ulcerative colitis	IVW	/	0.544–1.155	0.227
	AD	Crohn’s disease	IVW	/	0.890–1.720	0.205
Dong et al. (2023) [56]			IVW	0.976	0.955–0.997	0.029
Duan et al. (2021) [57]	Heart failure	AD	IVW	0.848	0.702–1.025	0.088
	AD	Heart failure	IVW	0.985	0.945–1.026	0.471
Li et al. (2022) [58]	AD	Apoplexy	IVW	1.02	0.99–1.05	0.286
	AD	Ischemic stroke	IVW	1	0.97–1.04	0.966
	AD	Cerebral hemorrhage	IVW	0.91	0.56–1.50	0.722
Tang et al. (2023) [59]	AD	Osteoporosis	IVW	0.918	0.805–1.047	0.201
Liu et al. (2022) [60]	AD	Obstructive sleep apnea	IVW	0.96	0.78–1.12	0.475
Zhang et al. (2020) [61]	Type II diabetes	AD	IVW	1.011	0.959–1.066	0.6921
	AD	Type II diabetes	IVW	1.047	1.000–1.096	0.049
Dybjer et al. (2023) [62]	Type II diabetes	AD	IVW	1.116	/	4.37 × 10^−1^
Litkowski et al. (2023) [63]	Type II diabetes	AD	Two-stage least squares (2SLS) MR	Non-Hispanic White participants: 1.06; non-Hispanic Black participants: 1.12	Non-Hispanic White participants: 1.02–1.09; non-Hispanic Black participants: 1.02–1.23	Non-Hispanic White participants: 6.84 × 10^−4^; non-Hispanic Black participants: 1.60 × 10^−2^
Luo et al. (2023) [64]	Type II diabetes	AD	IVW	1.01	0.98–1.04	0.56
Meng et al. (2022) [6]	Type II diabetes	AD	IVW	1.34	1.05–1.70	0.02
Pan et al. (2020) [65]	Type II diabetes	AD	IVW	1.02	0.97–1.07	0.52
Gao et al. (2023) [66]	Atrial fibrillation	AD	IVW	0.977	0.943–1.012	0.193
Kuo et al. (2022) [67]	Heart failure	AD	IVW	1.034	0.997–1.073	0.07
Wang et al. (2023) [68]	Hip osteoarthritis	AD	IVW	0.804	0.686–0.942	0.007
	Ankylosing spondylitis	AD	IVW	1.026	1.003–1.049	0.027
	AD	Rheumatoid arthritis	IVW	0.565	0.323–0.987	0.045
Yuan et al. (2024) [69]	Colorectal cancer	AD	IVW	0.846	0.762–0.929	/
	AD	Colorectal cancer	IVW	1.014	1.001–1.027	/
Zeng et al. (2024) [70]	Sepsis	AD	IVW	1.11	1.01–1.21	0.025
	AD	Sepsis	IVW	0.99	0.95–1.03	0.522
